# Supplementary material for: Movement-related coupling of human subthalamic nucleus spikes to cortical gamma
Source: eLife. 2020 Mar 11;9:e51956. doi: 10.7554/eLife.51956 (PMC7096181; doi:10.7554/eLife.51956)
Supplement: Transparent reporting form [file elife-51956-transrepform.pdf]

eLIFE
1st Floor
24 Hills Road
Cambridge CB2 1JP, UK
P 01223 855340
W elifesciences.org
T @elife

eLife's transparent reporting form

We encourage authors to provide detailed information *within their submission* to facilitate the interpretation and replication of experiments. Authors can upload supporting documentation to indicate the use of appropriate reporting guidelines for health-related research (see EQUATOR Network), life science research (see the BioSharing Information Resource), or the ARRIVE guidelines for reporting work involving animal research. Where applicable, authors should refer to any relevant reporting standards documents in this form.

If you have any questions, please consult our Journal Policies and/or contact us: editorial@elifesciences.org.

Sample-size estimation

- You should state whether an appropriate sample size was computed when the study was being designed
- You should state the statistical method of sample size computation and any required assumptions
- If no explicit power analysis was used, you should describe how you decided what sample (replicate) size (number) to use

Please outline where this information can be found within the submission (e.g., sections or figure legends), or explain why this information doesn't apply to your submission:

Since there are no previous studies on movement-related cortical gamma phase-STN spike coupling, we could not perform a formal power analysis. However, due to a relatively high signal-to-noise ratio of intracranial recordings, a small number of 9-12 patients is considered sufficient to detect robust effects in electrophysiological studies of the STN (e.g. Wessel et al., 2016; Fischer et al., 2017; Sharott et al., 2018). Our results are based on 28 unit recordings from 12 patients.

Wessel JR, et al. (2016) Surprise disrupts cognition via a fronto-basal ganglia suppressive mechanism. Nat. Commun. 7: 11195

Fischer P, et al. (2017) Subthalamic nucleus gamma activity increases not only during movement but also during movement inhibition. eLife. 6:e23947

Sharott, et al. (2018) Spatio-temporal dynamics of cortical drive to human subthalamic nucleus neurons in Parkinson's disease. Neurobiology of Disease. 112: 49-62

Since we did not perform a formal power analysis, this is not reported in the paper.

Replicates

- You should report how often each experiment was performed
- You should include a definition of biological versus technical replication
- The data obtained should be provided and sufficient information should be provided to indicate the number of independent biological and/or technical replicates

- If you encountered any outliers, you should describe how these were handled
- Criteria for exclusion/inclusion of data should be clearly stated
- High-throughput sequence data should be uploaded before submission, with a private link for reviewers provided (these are available from both GEO and ArrayExpress)

Please outline where this information can be found within the submission (e.g., sections or figure legends), or explain why this information doesn't apply to your submission:

The experiment was performed once intraoperatively in each patient.
Our biological replicates, i.e. "parallel measurements of biologically distinct samples that capture random biological variation" as defined by Blainey et al (2014, Nature Methods), are the 28 recordings of distinct single/multi-units from 12 patients.
The data is described in more detail in the Methods section "Task" and "Electrophysiological recordings".
Trials with RTs longer than 2s were excluded. (L766).
Trials containing artefacts in the ECoG signal were removed after visual inspection, and only recordings with at least eight contralateral grip trials (= technical replicates) were included (L796-797).

Statistical reporting

- Statistical analysis methods should be described and justified
- Raw data should be presented in figures whenever informative to do so (typically when N per group is less than 10)
- For each experiment, you should identify the statistical tests used, exact values of N, definitions of center, methods of multiple test correction, and dispersion and precision measures (e.g., mean, median, SD, SEM, confidence intervals; and, for the major substantive results, a measure of effect size (e.g., Pearson's r, Cohen's d)
- Report exact p-values wherever possible alongside the summary statistics and 95% confidence intervals. These should be reported for all key questions and not only when the p-value is less than 0.05.

Please outline where this information can be found within the submission (e.g., sections or figure legends), or explain why this information doesn't apply to your submission:

L951: Significance tests in plots showing multiple time- or frequency points were performed using a cluster-based permutation procedure for multiple-comparison correction (Maris and Oostenveld, 2007). The details of the procedure are described in the Methods section called "Cluster-based permutation procedure". No precise p-values are given for permutation test, since these vary slightly when repeating permutations.

L1022: Two-factorial 5 (bin) x 2 (effector side) repeated-measures ANOVAs were computed in SPSS (IBM Statistics SPSS 22, RRID:SCR_002865), to compare spike probabilities across the five phase-bins. Q-Q plots of the residuals were visually inspected to exclude strong deviations from normality. If the sphericity assumption was violated, Huynh-Feldt correction was applied. Subsequent pairwise comparisons were performed using t-tests or Wilcoxon signed-rank tests if the normality assumption (assessed by Lilliefors tests) was violated.

L1034: The angle difference between contra- and ipsilateral gripping was tested against zero by computing confidence intervals (*circ_confmean* function from the *circ_stats* toolbox (Berens, 2009)).

L1041: We also tested when exactly the offset of the preferred phase during contra- and ipsilateral gripping would approximate 180° degrees with a V-test (*circ_vtest* function from the *circ_stats* toolbox (Berens, 2009)). A V-test examines the phase differences for circular uniformity, similarly as a Rayleigh test, but is more powerful because a mean difference – in this case 180° – can be specified.

L1070: To compare the slopes of the increasing high-frequency activity between different cortical sites, t-scores of the slope of a linear regression line fitted to the ascending phase of the gamma cycle (between -π:0 rad, and between -0.75π:-0.25π rad to assess only the steepest part) were computed for each recording and then evaluated on the group level.

Where n was not equal to 28, we explicitly stated it.

(For large datasets, or papers with a very large number of statistical tests, you may upload a single table file with tests, Ns, etc., with reference to sections in the manuscript.)

Group allocation

- Indicate how samples were allocated into experimental groups (in the case of clinical studies, please specify allocation to treatment method); if randomization was used, please also state if restricted randomization was applied
- Indicate if masking was used during group allocation, data collection and/or data analysis

Please outline where this information can be found within the submission (e.g., sections or figure legends), or explain why this information doesn't apply to your submission:

This was not a clinical study. We had only one experimental group.
All participants performed the same experimental tasks and within-subject comparisons were performed relative to baseline or permutation data.

Additional data files ("source data")

- We encourage you to upload relevant additional data files, such as numerical data that are represented as a graph in a figure, or as a summary table
- Where provided, these should be in the most useful format, and they can be uploaded as "Source data" files linked to a main figure or table
- Include model definition files including the full list of parameters used
- Include code used for data analysis (e.g., R, MatLab)
- Avoid stating that data files are "available upon request"

Please indicate the figures or tables for which source data files have been provided:

We have uploaded the data and the code (including the functions to run the cluster-based permutation statistics) with which one can generate the time-frequency figures in the main manuscript and in the supplementary figures (Fig. 2, 3, S5 and S7).
